# Rise of Clinical Studies in the Field of Machine Learning: A Review of Data Registered in ClinicalTrials.gov

**DOI:** 10.3390/ijerph18105072

**Published:** 2021-05-11

**Authors:** Claus Zippel, Sabine Bohnet-Joschko

**Affiliations:** Chair of Management and Innovation in Health Care, Faculty of Management, Economics and Society, Witten/Herdecke University, 58448 Witten, Germany; claus.zippel@uni-wh.de

**Keywords:** machine learning, digital health, registry analysis, ClinicalTrials.gov, device regulation

## Abstract

Although advances in machine-learning healthcare applications promise great potential for innovative medical care, few data are available on the translational status of these new technologies. We aimed to provide a comprehensive characterization of the development and status quo of clinical studies in the field of machine learning. For this purpose, we performed a registry-based analysis of machine-learning-related studies that were published and first available in the ClinicalTrials.gov database until 2020, using the database’s study classification. In total, *n* = 358 eligible studies could be included in the analysis. Of these, 82% were initiated by academic institutions/university (hospitals) and 18% by industry sponsors. A total of 96% were national and 4% international. About half of the studies (47%) had at least one recruiting location in a country in North America, followed by Europe (37%) and Asia (15%). Most of the studies reported were initiated in the medical field of imaging (12%), followed by cardiology, psychiatry, anesthesia/intensive care medicine (all 11%) and neurology (10%). Although the majority of the clinical studies were still initiated in an academic research context, the first industry-financed projects on machine-learning-based algorithms are becoming visible. The number of clinical studies with machine-learning-related applications and the variety of medical challenges addressed serve to indicate their increasing importance in future clinical care. Finally, they also set a time frame for the adjustment of medical device-related regulation and governance.

## 1. Introduction

### 1.1. Background

Before medical innovations can be implemented in daily clinical routine, it takes more than a decade from research and development to market approval [1,2,3]. In this translation phase, a multitude of challenges and specifications have to be overcome so that a device can successfully be brought to the market, from patient recruitment, data consolidation and fragmented infrastructures to regulatory hurdles and (start-up) financing of research costs [4,5]. Examining the literature, it is noticeable that so far, there are hardly any data on the specific translation process of medical–digital applications that are increasingly being developed and that promise great benefits and potentials for health prevention, diagnostics, and therapy [6,7,8,9,10].

### 1.2. Research Motivation and Objective

Against this background, it was our aim to explore the development and current translation status of medical–digital applications in the field of machine learning (ML), a sub-area of artificial intelligence in which computer algorithms and statistical models are trained based on large datasets to independently link and predict abnormalities and correlations in a self-learning manner [11,12,13,14,15]. We focused on ML, as there are already a wide range of ML-based approaches and innovative developments for health care reported in the literature, from image diagnostics and processing [16,17,18,19,20], personalized medicine and genomics [21,22,23] to clinical data analysis for decision support and training in surgery, therapy planning or patient management [24,25,26,27,28].

In view of the research question, we decided to analyze study register data as they offer a glance into the research pipeline of universities, university clinics and research institutions as well as pharmaceutical, medical device, and biotech companies, and thus, provide first insights into the clinical translation process of ML-related applications and software. This registry-based approach also allows us to cluster and identify fields with increased research and investment that might be of clinical significance in the next decade. Considering legislative delays, our results may support health decision- and policymakers struggling with challenges in the regulation and governance of ML-applications [29,30,31].

## 2. Materials and Methods

### 2.1. Data Acquisition and Processing

For our study, we used datasets from ClinicalTrials.gov, one of the most comprehensive databases for clinical studies worldwide with over 360,000 planned, ongoing and completed clinical studies published at the time of access [32,33,34,35]. The register is freely accessible via https://clinicaltrials.gov [36]. For each study, (i) a given set of study characteristics is compulsory, and (ii) study-specific details are requested, using free text fields, such as title or individual short description. The ClinicalTrials.gov database and methodological approach have already been chosen frequently in other research studies to characterize study populations and trends in clinical care and research [37,38], for example in the areas of medical imaging [39,40,41], rare diseases [42] or oncology [43,44].

**Figure 1:**
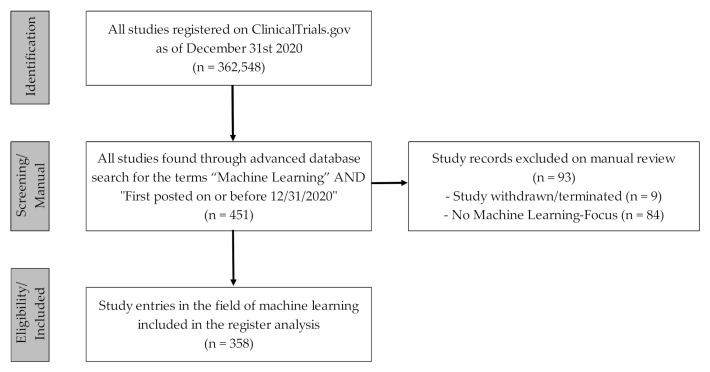
Flowchart for the selection procedure of the ML-related clinical study entries considered for the quantitative registry analysis. Source: Own figure based on the evaluation of the ClincalTrials.gov dataset [36].

In view of the research question, the “advanced search function” was used to filter the register data records for which “Machine Learning” (a MeSH term introduced in 2016, [12]) had been entered in the report form and which were published by the end of 2020 (search term: “Machine Learning”|First posted on or before 31 December 2020). The dataset was retrieved on 7 January 2021 and exported in CSV file format [36]. In a second step, the authors scanned the dataset and included all study entries that clearly focused on the use or testing of ML-based algorithms, approaches or applications in a clinical setting. Entries on clinical trials that, according to the reporting party, were “withdrawn” or “terminated” or clearly did not primarily focus on the use of ML-related approaches or applications in clinical care were excluded from the study. In order to be able to filter and subgroup the studies in detail, the authors scanned the free text information of the study entries. Figure 1 shows the methodical procedure for the selection process of the study dataset considered for the register data analysis in the form of a flowchart.

### 2.2. Data Evaluation and Analysis

In order to provide an overview of the development and status quo of ML-related software approaches and applications in the clinical setting, the study entries were sorted in ascending order according to the date of which the study record was first available on ClinicalTrials.gov. Furthermore, common standardized study parameters, such as study type, recruitment status, age group or funding source, were evaluated [45]. In order to achieve a more in-depth characterization of the dataset, the authors scanned, evaluated and subcategorized the study entries according to further parameters, such as recruiting country, academic/industry sponsor or clinical study-initiating medical specialty/field. Further free text information, such as intervention arms, inclusion criteria or end points of the trials, were not part of the study.

In view of the explorative nature of the study objective, we evaluated the registry dataset descriptively. One-dimensional frequency distributions (absolute, relative) were determined for the analyzed study characteristics. The development of the published studies per year over time was shown graphically using a bar chart, and the description of all other parameters was summarized in tables. The quantitative acquisition, processing and statistical evaluation of the dataset was carried out, using Microsoft Excel^®^ software for Microsoft Windows^®^.

## 3. Results

### 3.1. Registration of ML-Related Studies over Time

**Figure 2:**
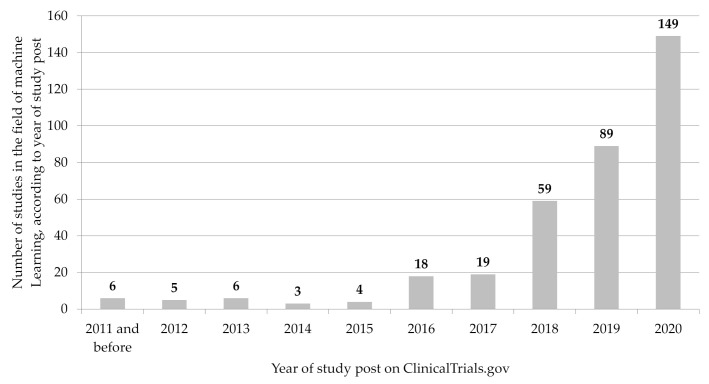
Number of clinical studies related to ML by year of publication on ClinicalTrials.gov (*n* = 358). Source: Own figure based on the evaluation of the ClincalTrials.gov dataset [36].

For our study, *n* = 358 study entries in the field of ML were included (see Figure 1). Sorted by year of first publication in the ClinicalTrials.gov register, a continuous rise in ML-related study entries could be seen since 2015, with a particularly significant increase between 2019 and 2020, from *n* = 89 to *n* = 149 posted studies (see Figure 2).

### 3.2. Medical Field of Application

**Figure 3:**
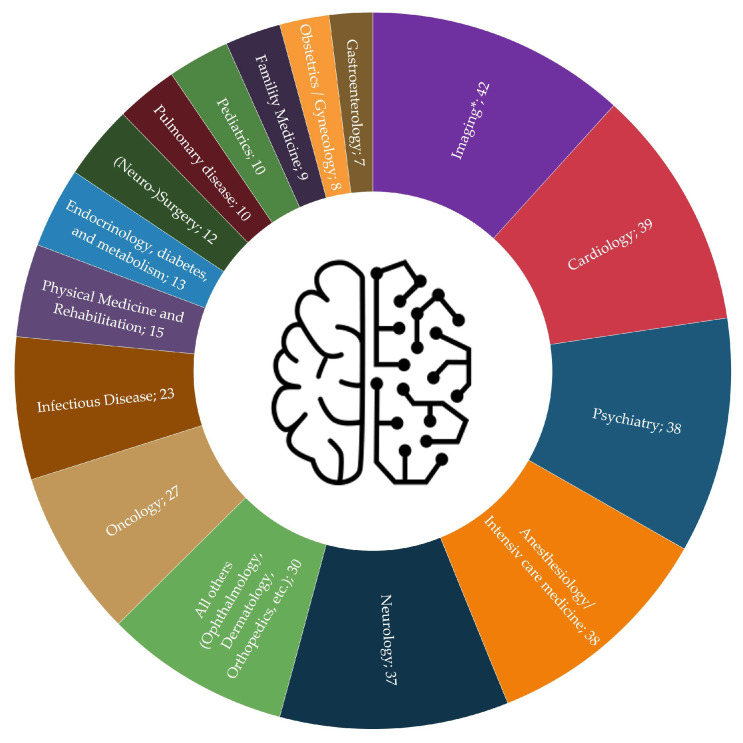
Study entries in the field of ML by study-initiating medical specialty/field (*n* = 358). Source: Own figure based on the evaluation of the ClincalTrials.gov dataset [36]. * Dianostic Radiology/Biomedical Imaging, Radiation Oncology, Nuclear Medicine.

The registered studies focused on a broad spectrum of different topics from a wide range of medical specialties. The majority of the posted studies in the field of machine learning was initiated by experts from the field of imaging (diagnostic radiology, nuclear medicine, radiation oncology; 12%), followed by cardiology, psychiatry, anesthesia/intensive care medicine (all 11%), neurology (10%), medical oncology (8%) and infectious disease medicine (6%) (see Figure 3). The latter mainly included studies that were published in 2020 on COVID-19-related issues.

### 3.3. Patient Recruitment and Study Organization

About half of the listed clinical studies were open (55%) or closed (45%) for patient enrollment. A total of 27% of the studies had already completed the recruitment phase. The vast majority of studies (98%) did not yet have any results. A total of 80% of the studies in the dataset were single-center, 13% multi-center studies. Seven percent could not be classified because of missing information (see, for this and the following, Table 1). Of the studies, 96% were national and 4% international. Of these, by far the most studies had a last recruiting location in the U.S.A. (40%), followed by China (9%), the United Kingdom (8%), Canada (6%), France (5%), Switzerland and Germany (each 4%). Across all study entries, and with a view to the major global regulatory regions, most of the published studies recruited patients in a country in North America (47%), followed by Europe (37%) and Asia (15%; other 6%).

In 82% of the studies, a university (hospital) and/or research institution was named as the organization/person responsible for the study (so-called “lead sponsor”), and in 18%, an industrial company. The majority of trials (88%) was (co-) funded by individuals, universities or organizations themselves, 24% of trials were (co-) funded by the industry and 5% had a public (government) sponsorship.

### 3.4. Study Type and Design

Of the *n* = 358 clinical studies categorized, around two thirds (64%) were reported as observational studies and around one third (36%) as interventional studies (see, for this and the following, Table 2). Among the observational studies, the majority of the studies were designed as prospective cohort studies. The majority of the interventional studies was open label/non-masked and single-armed. Over 90% of the studies planned to enroll (elderly) patients of both genders.

## 4. Discussion and Conclusions

Recent improvements and innovative approaches in the field of artificial intelligence promise high potential for the diagnosis and treatment of patients [46,47,48,49]. The sub-area of ML in which self-learning algorithms (such as convolutional neural network, random forest, support vector machine, etc. [50,51,52]) are trained on large datasets and used to make predictions independently when exposed to new data, is particularly advancing [11,13,14,17,19,20]. More and more research is showing that newly developed algorithms can process specialized tasks just as well as experienced health professionals or can increase their efficiency and performance in daily care [53,54,55,56]. A crucial factor for the successful development of ML-based software and assistance systems is—besides medical and technological expertise—in particular, the testing and use of these applications in daily clinical routine [57,58]. With this in mind, it was our goal to find out more about the recent development and status of the clinical translation of ML-related software and applications into the clinical setting. The translation and market approval of ML-based algorithms represent a major challenge in terms of legislation and regulation. Using the example of register data, the results show how dynamically this area is developing across medical disciplines. As a result, questions about governance and clinical testing will have to be answered in the near future (cf. for example [29,30,31]). In the following sections, we will summarize the main results of the registry data analysis on ML-related clinical studies, discuss this with reference to the regulatory environment and point out the methodological limitations of the study.

### 4.1. Studies in the Field of ML

The study data show that the number of ML-related studies in ClinicalTrials.gov has increased continuously from year to year since 2015, with a particular increase between 2019 and 2020 (see Figure 1). From a methodological point of view, it should be noted that the MeSH term “machine learning”, which was crucial for the study search in the registry database, was introduced in 2016 by the U.S. National Library of Medicine [12]. This could have influenced the search and selection procedure (especially for the period before 2015), as this MeSH term was probably only systematically reported and checked as a quality control review criteria for the clinical study registration from this point in time [59]. For the last few years, however, a visible increase in the number of published studies can be determined. This could be an indicator for the growing potential that is associated with the use and application of ML-related software/algorithms for medical care and research.

In addition, it was found that the majority of the analyzed studies in the field of ML were initiated and led by (university) hospitals or academic/research institutions (82%) and were (co-) financed from university (88%) or public/government funds (5%) (see Table 1). Among the academic institutions, most of the registered studies were reported by the Mayo Clinic (U.S.), Maastricht University Medical Center (NL), Sun Yat-Sen University (CN) and University of California (U.S.). In this context, the authors assume, that the number and proportion of academically initiated ML-related studies is likely to be underestimated here since the sponsor or PI in some cases does not necessarily have to register an academic study in a database such as ClinicalTrials.gov. This is especially the case for studies in the preclinical development stage or if only retrospective data are used. In comparison, fewer studies were initiated (18%) or (co-) financed (24%) by an industry sponsor. The proportion of studies with an industrial study sponsor is (still) relatively low, compared to other publications on ClinicalTrials.gov study data. For example, a cross-sectional analysis by Ross et al., published in 2009, showed a proportion of 40% in studies with industry sponsors [38] and a study by Bell and Smith from 2014 on over 24 thousand clinical studies on rare and non-rare conditions showed a proportion of more than 30 percent [42].

Among the industry sponsors were several comparatively small companies and start-ups with a focus on the development of algorithms in medicine (e.g., Dascena^®^ and Eko Devices^®^). In general, it can therefore be assumed that the ML-related approaches reported were still mainly initiated and used in an academic/research context but could gradually be transferred to clinical translation and early clinical study development phases with increasing support from the industry, which sees investment potentials in this area.

Moreover, the analyzed studies were initiated from a variety of different medical fields and disciplines (Figure 3). Looking at the dataset, it could be seen that the ML-related approaches in the clinical studies used different types of training data. This included image data (e.g., in radiomics studies), sensor data (e.g., ECG signals), video data, text data and audio data (e.g., monitor audio signals). Furthermore, the registered studies used a wide range of different types and approaches of ML algorithms, such as (un-) supervised or reinforced learning. In order to illustrate this heterogeneity, we show selected study approaches from different medical application areas and fields. We hereby focus on advanced clinical studies for which the recruitment phase was reported as completed and at least one scientific publication was available.

- Blomberg et al. reported to analyze whether a ML-based algorithm could recognize out-of-hospital cardiac arrests from audio files of calls to the emergency medical dispatch center (NCT04219306, [60]);
- Jaroszewski et al. wanted to evaluate a ML-Driven Risk Assessment and Intervention Platform to increase the use of psychiatric crisis services (NCT03633825; [61]);
- Mohr et al. stated to evaluate and compare a smartphone intervention for depression and anxiety that uses ML to optimize treatment for participants [NCT02801877; [62]);
- Nieman et al. conducted a study to investigate the diagnostic performance of ML-based, coronary computed, tomography–angiography-derived fractional flow reserve (NCT02805621; [63,64,65]);
- Putcha et al. performed a study on a ML-based approach to discover signatures in cell-free DNA to potentially improve the detection of colorectal cancer (NCT03688906; [66,67].

In summary, the results of the registry data analysis show that the registered studies in the field of ML were very heterogeneous, both from an organizational and study design perspective. Against this background, it would make sense to carry out further (especially multivariate) sub-evaluations of the dataset for selected study groups, for example, with large cohort radiomics studies, etc. Finally, it should be noted that the imaging disciplines in particular are involved in many studies, both as a study-initiating discipline and as a clinical partner, for example, for CT, MRI or PET scans. Since only the respective, study-initiating department was focused on for the register analysis, it can be assumed that the proportion of ML-related studies in which imaging experts are centrally integrated is significantly higher than the 12% shown in Figure 3.

### 4.2. Regulatory Framework and Aspects

With regard to the dataset, it is essential to point out, from a regulatory point of view, that the posted studies in the field of ML always address software that, in many cases, functions or is used (directly) in connection with a medical device. This is of central importance since from a regulatory point of view, software is considered a medical product in many regulatory areas, such as the U.S. or the European Union [68], and is, therefore, subject to the associated regulatory requirements, such as conformity assessment, registration, clinical evaluation or post-market surveillance [69]. In the EU, for example, software is considered a medical device according to the European Medical Device Regulation (MDR), which will come into force in May 2021, “when specifically intended by the manufacturer to be used for one or more […] medical purposes […], independent of the software’s location or the type of interconnection between the software and a device” [70]. The risk classification is based on the diagnostic and therapeutic intension of the software from risk classes I (lowest risk class) to III (highest risk class).

In this context, it should be pointed out that for ML-related software, primarily the general regulatory requirements for software apply and that there are hardly any laws or harmonized standards for the specific use of ML-software and applications in healthcare. With this in mind, it is of great interest that the U.S. Food and Drug Administration (FDA) has published a discussion paper on “Artificial Intelligence/Machine Learning (AI/ML)-Based Software as a Medical Device (SaMD) Action Plan”, which is continuously updated and currently making proposals with regard to the following areas:

- Tailored regulatory framework for AI/ML-based SaMD;
- Good machine-learning practice;
- Patient-centered approach, incorporating transparency to users;
- Regulatory science methods related to algorithm bias and robustness;
- Real-world performance [71].

In view of the increasing amounts of clinical studies in the field of ML (Figure 1), it will be interesting to see how the regulatory framework will adapt, worldwide, to AI- and ML-related software and applications as well as the specifics associated with them. Aspects that have not yet been clarified, such as changes in ML-related software over time due to changing datasets, should be of particular interest. In the literature, suggestions are increasingly being submitted and discussed [30,72], both on general regulatory aspects [29,73,74] and on device- or subject-specific features, e.g., in view of medical imaging [75,76].

In addition, it becomes clear how important it will be in the future to pool patient data for clinical studies in the field of machine learning across multiple locations. The reason for this is that access to large amounts of data will be essential for the further development of the approaches in prospective clinical studies. An example of how this could work in view of strict data protection requirements is shown by the Joint Imaging Platform for Federated Clinical Data Analytics for the application of medical algorithms across study sites in the field of medical imaging [77].

### 4.3. Methodological Notes

The evaluation of registry data from ClinicalTrials.gov enables a broad and detailed analysis of a multitude of systematically collected, study-specific entries of high quality over a period of time. However, a number of limitations to this study approach need to be noted. Firstly, a method-inherent error of this approach is that the register dataset only represents a subset of all initiated ML-related studies around the globe. The reasons for this are that in some cases, the PI or sponsor does not necessarily have to register the study (see Section 4.2) or may as well choose a different registry to list the study accordingly [78,79,80]. In this context, it should also be pointed out that data and information specifically relating to research in the field of machine learning are also published in other digital archives or specific registers and research platforms, such as the platform of the Association for Computing Machinery (ACM) or the Institute of Electrical and Electronics Engineers (IEEE). This both illustrates the importance of harmonizing the fairly large number of registries and archives to prospectively create (also linguistically) more uniform data, a process that is focused on by projects such as the “Research Data Alliance” or the “Open Data Institute”. Secondly, the registry search only took into account register entries in which the search term “machine learning” was explicitly specified in the study title or free text. Since the use of study-specific MeSH terms when registering studies in ClinicalTrials.gov is recommended but not mandatory, it can be assumed that studies that used other MeSH terms or were registered with terms related in taxonomy were not taken into account for the dataset. This may well lead to the fact that the actual number of ML-related clinical studies published, and thus the clinical development in this field, is probably underestimated. Thirdly, common limitations of clinical registry (meta-) data analyses apply, which can lead to inaccuracy and inconsistencies, and thus may impair the data quality. This includes, in particular, incorrect or not-at-all answered sections of the registry form. In addition, the study text information (some of which vary in scope and content) can be interpreted differently, which could reduce the validity of the results [37,39,40,41,42,43,44,45]. Fourthly, the subgrouping of studies into medical specialties was not always clear; for example, when experts from two or more medical specialties were involved. In order to avoid this methodological problem, the medical specialty of the PI responsible for the trial and named in the study entry was used for subgrouping in case of doubt. As a result, medical specialties that are often involved in ML-related studies but tend to initiate fewer studies as the lead medical specialty were probably counted less (e.g., (neuro-) pathology [81]). Fifthly, it has to be assumed that since ClinicalTrials.gov is an American registry, there is a disproportionately high number of registered clinical trials conducted in North America. Our study results strongly support this hypothesis, seeing that the vast majority of studies included those recruited in the U.S.A. and Canada (see Table 1). This may possibly lead to distortions in comparison to the status and characteristics of ML-related trials in other regions, such as Europe or Asia.

In view of the limitations, the present study cannot represent a complete, detailed picture of the status quo. However, since ClinicalTrials.gov is by far the biggest and most renowned registry for clinical trials, the authors conclude that this approach allows a good first overview on the current status of clinical development and translation of ML-based approaches and applications in health care. This could provide an impetus for decisionmakers in healthcare facilities and policy as well as regulatory discussions.

## 5. Summary for Decisionmakers

- In recent years, an increasing number of ML algorithms have been developed for the health care sector that offer tremendous potential for the improvement of medical diagnostics and treatment. With a quantitative analysis of register data, the present study aims to give an overview of the recent development and current status of clinical studies in the field of ML.
- Based on an analysis of data from the registry platform ClinicalTrials.gov, we show that the number of registered clinical studies in the field of ML has continuously increased from year to year since 2015, with a particularly significant increase in the last two years.
- The studies analyzed were initiated by a variety of medical specialties, addressed a wide range of medical issues and used different types of data.
- Although academic institutions and (university) hospitals initiated most studies, more and more ML-related algorithms are finding their way into clinical translation with increasing industry funding.
- The increase in the number of studies analyzed shows how important it is to further develop current medical device regulations, specifically in view of the ML-based software product category. The recommendations recently presented by the FDA can provide an important impetus for this.
- Future research with trial registry data might address sub-evaluations on individual study groups.

## Figures and Tables

**Table 1:** Recruitment and organizational parameters of the included ML-related trials from the ClinicalTrials.gov registry (*n* = 358).

	Absolute (*n*)	Relative (%) *
Overall study status *		
***Patient recruitment***		
Open	198	55
Not open	160	45
***Recruitment status***		
Not yet recruiting	64	18
Recruiting	134	37
Enrolling by invitation	15	4
Active, not recruiting	22	6
Suspended	5	1
Completed	95	27
Unknown status	23	6
***Study results***		
Studies with results	6	2
Studies without results	352	98
**Organization/Cooperation**		
***Number of study locations***		
Single study location	288	80
Multiple study locations	46	13
Not clear	24	7
***National/International***		
National	345	96
International	13	4
Study location/Recruiting country **		
The United States of America	144	40
China	34	9
The United Kingdom	28	8
Canada	23	6
France	18	5
Switzerland	14	4
Germany	13	4
Israel	12	3
Spain	12	3
Netherlands	11	3
All others (Republic of Korea, Italy, Belgium, etc.)	67	19
**Lead sponsor**		
University/Hospital	292	82
Industry	66	18
Funding Sources **		
Industry	86	24
All others (individuals, universities, organizations)	314	88
Government agencies	19	5
National Institutes of Health (NIH) ***	*11*	*3*
Other U.S. Federal Agency ***	*8*	*2*

* Sum partly ≠ 100 due to rounding; ** More than one choice possible; *** Subcategories in italics; Source: Own table based on the evaluation of the ClincalTrials.gov dataset [36].

**Table 2:** Study type and study design specific parameters of the included ML-related clinical trials from the ClinicalTrials.gov registry (*n* = 358).

	Absolute (*n*)	Relative (%) *
**Population studied**		
Age group **		
Included children	74	21
Included adults	341	95
Included older adults (age > 65 year)	320	89
***Gender of participants***		
Both	333	93
Female only	20	6
Male only	5	1
**Study type and design**		
Observational Studies ***	***230***	***64***
*Observational Model*		
Cohort	154	43
Case-Control	26	7
Case-Only	26	7
Other	24	7
*Time Perspective*		
Prospective	140	39
Retrospective	57	16
Cross Sectional	17	5
Other	16	4
Interventional Studies ***	***128***	***36***
*Allocation*		
Randomized	66	18
Non-Randomized	17	5
N/A	45	13
*Intervention Model*		
Single Group Assignment	48	13
Parallel Assignment	69	19
Other (crossover, sequential, etc.)	11	3
*Masking/Blinding*		
None (Open Label)	77	22
Masked	51	14
*Single (Participant or Outcomes Assessor)*	*19*	*5*
*Double or triple*	*32*	*9*
*Primary purpose*		
Diagnostic	37	10
Treatment	26	7
Prevention	12	3
Supportive Care	11	3
Other	42	12
Intervention/treatment type **		
Behavioral	40	11
Device	86	24
Diagnostic Test	77	22
Drug	17	5
Procedure	13	4
Other	155	43

* Sum partly ≠ 100 due to rounding; ** More than one choice possible; *** Subcategories in italics; Source: Own table based on the evaluation of the ClincalTrials.gov dataset [36].

## Data Availability

For the study, data from the website ClinicalTrials.gov were used [36]. The registry for clinical studies is available online https://clinicaltrials.gov (accessed on 7 January 2021).
